# Spectrogram Analysis of Complete Dentures with Different Thickness and Palatal Rugae Materials on Speech Production

**DOI:** 10.1155/2015/606834

**Published:** 2015-03-26

**Authors:** Hamada Zaki Mahross, Kusai Baroudi

**Affiliations:** ^1^Department of Removable Prosthodontics, College of Dentistry, Al-Azhar University, Cairo, Egypt; ^2^Department of Restorative Dental Sciences, Alfarabi Colleges, Riyadh, Saudi Arabia

## Abstract

*Objective*. To investigate the influence of reproduction of different thickness and palatal rugae materials on complete dentures speech using Computerized Speech Lab (CSL) (spectrogram). *Materials and Methods*. Three completely edentulous male patients (aged 50–60 years) were selected for reading a paragraph. Twelve upper dentures were constructed, four for each patient. The patients' speech groups were divided into five groups, Group I: patients without dentures; Group II: patients rehabilitated with conventional acrylic dentures; Group III: patients with conventional acrylic dentures with rugae reproduction; Group IV: patients with dentures with metallic framework of minimal thickness and direct ragged metallic palatal surface at rugae area; Group V: patients with dentures with palatal rugae constructed from resilient acrylic resin material with thickness less than conventional denture. Speech samples were recorded after insertion of each denture for groups using Computerized Speech Lab (CSL) (spectrogram). The sounds selected were lingopalatal /s/z/sh/t/d/ and /l/. *Results*. Group III produced high mean significant difference with /sh/t/ sound. For Group IV, the difference was noticed with /s/z/sh/t/ and /d/ sounds, while for Group V the difference was shown with /z/l/ sound (*P* < 0.05). *Conclusion*. It is recommended to reproduce the rugae area in complete denture because the phonetic quality of complete denture with rugae is superior to the conventional denture.

## 1. Introduction

Speech is essential to human activity as it is an important function of the stomatognathic system, which uses the oral cavity as a part of the vocal apparatus. The voice is produced in the larynx with the aid of the vocal cords vibrating due to the expiratory airflow. The frequency of the fundamental laryngeal tone is dependent on the vocal cord tension, which then modulated in the resonance cavities and their shape conditioning the vowel formants [1, 2].

The speech articulatory organs include the tongue, palate, alveolar processes, gums, teeth, and lips. The teeth, alveolus, and palate are static components of speech articulation whereas tongue, lip, and velum are dynamic components. Therefore, phonetics must be considered with mechanics and esthetics as the cardinal factors contributing to the success of the dental prosthesis [2, 3].

Complete loss of teeth can cause persistent speech disorders by altering dental articulation areas that will severely reduce the quality of speech; particularly the alteration of frontal maxillary morphology leads to impairment of speech production [4].

Removable complete dentures can partly solve this problem. However, they disturb speech production themselves as they restrict the flexibility of the tongue, narrow the oral cavity, and alter the articulation areas of the palate and teeth [5, 6].

Dentures should be made to enable the patient to produce voice and speech without deficiencies. About 25% of patients in clinical dentistry are considered to suffer from temporary or permanent changes in articulation due to the application of removable dentures. Articulatory errors may be due to denture factors like altered vertical dimension, size, and position of the teeth, thickness, and contour of the denture base [1, 2].

Accurate approximation of palatal contours of a maxillary complete denture to a patient's tongue can improve speech intelligibility, if other factors such as tooth position, occlusal plane, and occlusal vertical dimension are satisfactory [7].

Pound, in 1950, stated that anterior palatal region plays an important role in pronunciation of consonants. He termed it as “playground” of tongue because 90% of tongue's rapid manipulation while talking was restricted to this area and area lingual to lower anterior teeth [8].

The artificial palatal vaults of maxillary dentures often have a concave shape. In contrast, natural palatal vaults are of convex shape in the alveolar region. So the palatal vaults of maxillary dentures should be shaped like natural ones to facilitate correct pronunciation and the quality of speech sound production improved within the first week after insertion of new dentures [9].

The most important instruments for the investigation of speech sound production are the trained hearing of a speech and language professional, spectral analysis, and palatographics [4, 10, 11].

Spectral analysis has been used to examine the effects of dental prostheses on speech sound production. With the use of spectral analysis, a sound event can be split into three dimensions: frequency, amplitude, and chronologic sequence. The data obtained can be visualized in 2-dimensional Cartesian coordinates with the color or grey shade as the third dimension. Although less common, a 3-dimensional profile representation can be used instead [4, 5, 11, 12].

Studies on speech sound production may have a clinical impact because many patients attach great importance to undisturbed speech sound production after dental treatment and Heyink et al. found that 28 (21%) of 131 denture wearing individuals from an elderly population had speech problems. Speech production has a significant effect on patients' general satisfaction with dentures [13].

The aim of this study was to investigate the influence of reproduction of different palatal rugae materials and thickness on complete dentures speech using spectrogram.

## 2. Materials and Methods

Three completely edentulous male patients, aged 50–60, were selected from the Outpatient Clinic of Prosthodontics Department, Faculty of Dental Medicine, Al-Azhar University (ethical approval number 589/2012). The selected patients must be free from any systemic diseases or any dental problems and must be able to read in a regular manner for reading the paragraph. Patients consent was obtained.

Twelve complete upper dentures were incorporated in this study; four upper complete dentures with different palatal rogue changes for each patient were constructed. The patients' speech records in this study were divided into five groups, Group I: patients without denture, Group II: patients with upper acrylic denture having polished anterior palatal surface without rugae reproduction in usual thickness (2–2.5 mm), Group III: patients with conventional upper acrylic denture with rugae reproduction on its polishing surfaces in usual thickness (2–2.5 mm), Group IV: patients with metallic upper denture base of minimal thickness (0.5–1 mm) and direct ragged metallic palatal surface at rugae area, and Group V: patients with upper denture having palatal rugae constructed from resilient acrylic resin material with thickness less than conventional denture (1.5–2 mm).

The dentures in Groups III, IV, and V were made by duplicating the denture previously constructed to Group II to preserve the same tooth position, occlusal plane, vertical dimension, and denture base thickness for each patient. After that, the duplicated denture was used as trial denture as the rugae reproduction was performed.

### 2.1. Reproduction of the Rugae Area

Two maxillary alginate impressions were made for a patient with prominent palatal rugae. The first impression was poured with stone to be used as a palatal rugae replica. The second impression was performed as the area of rugae in the impression was poured with softened wax to make a palatal rugae template. The wax was removed from the impression and sealed on the polishing surface of the trial duplicated denture base at the area of rugae.

The trial denture was processed in the usual manner, finally finishing and polishing the final denture with its rugae.

The patient adaptation period must be about two weeks to avoid any mistake of new denture problems. According to Inukai et al. the speech adaptation to new complete denture normally takes place within 2 to 4 weeks after insertion [14].

### 2.2. Methods of Measurements

Speech samples were recorded after insertion of each denture for groups using Computerized Speech Lab (CSL) (spectrogram) (CSL Kay Elemetrics Model 4300, USA) in Phoneatric Unit, ENT Department, Specialized Ain-Shams Hospital.

After the period of adaptation, the sound recordings were performed in a special room phonically isolated with the patient speaking in a sensible microphone. A microphone produces a time-varying electrical voltage that is proportional to the increase or decrease in local pressure that constitutes sound. This continuous time-varying voltage is an electric analog of the acoustic signal [15]. The patients were asked to sit in upright position to allow reading of a passage in comfortable amplitude and pitch. Distance from microphone is about 10 cm to avoid a lot of stochastic noise.

The set unit CSL consists of external module with high speed dual channel, the plug-in board with 2 high speed digital signal processing integrated circuits, high quality microphone, and software version 3.1 and all patients were subjected to analysis of their signal after it is captured using sampling rate of 20 KHz and bandwidth is 300. The continuous electric signal can be converted to a digital representation suitable for manipulation by a computer. Computers equipped with the proper hardware can convert the analog voltage variations into digital sound waveforms by a process called analog-to-digital conversion.

The higher the sampling rate, the better the sound quality, but the more the bits required. There is a direct relationship between the accuracy of quantization and the number of bits required [4, 15].

The tested sounds selected were /S/ unvoiced linguodental fricative; /Sh/ unvoiced linguoalveolar fricative; /z/ voiced linguoalveolar fricative; /t/ unvoiced linguoalveolar stop; /d/ voiced linguoalveolar stop; /l/ voiced linguoalveolar stop.

These sounds were selected in a paragraph to avoid the patient trials to adjust himself for optimum utterance. The spectral analysis was not done on the whole word of the paragraph but only on word fragments that permit perception of these alterations as /s/sh/z/t/d/ and /l/ sounds. Breaking the words into sounds was performed for each patient in order to achieve more profound study of individual phonetic characteristics and a higher quality of studied phenomena.

The spectral analysis of the taken signal was measured by the maximum energy (frequency, HZ) for /s/, /sh/, and /z/ sounds. However, for /t/d/ and /l/ sounds, it was measured by time interval (m/sec.) because they have less voice onset time for adaptive patient speak. The data were obtained using vertical and horizontal cursors on the screen.

### 2.3. Statistical Analysis

Data were statistically analyzed by one-way ANOVA followed by Tukey's post hoc test at the significance level of *α* = 0.05.

## 3. Results

Data of the tested groups was presented as mean and standard deviation (SD) values (Table 1).

**Figure 1:**
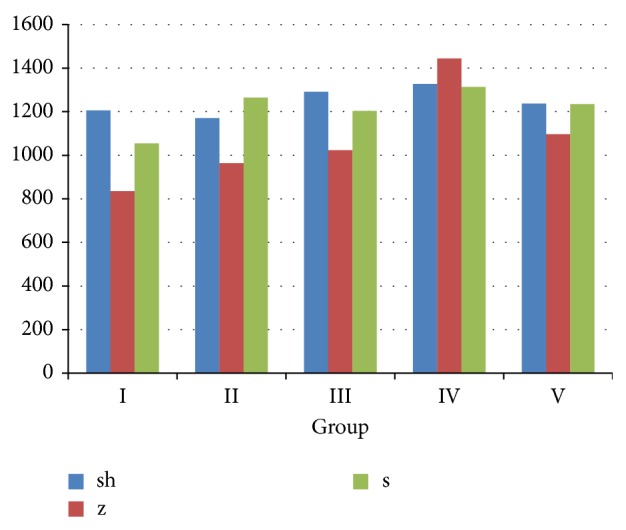
Comparison between the studied groups according to /sh/z/t/ sounds measured by the maximum energy (frequency, HZ).

**Figure 2:**
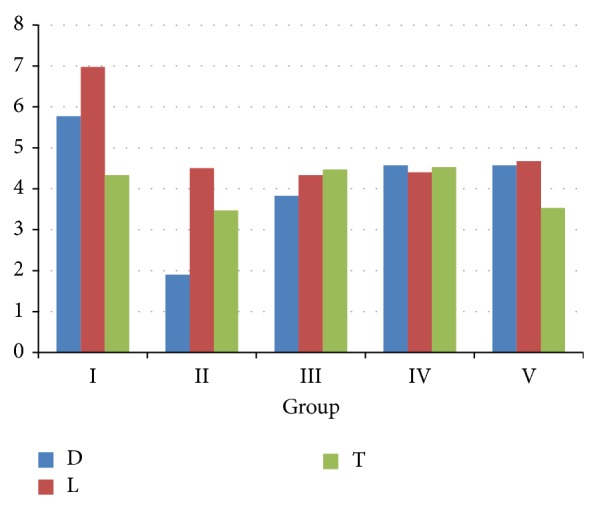
Comparison between the studied groups according to /d/l/t sounds measured by time interval (m/sec.).

The collected data for /s/ sound in five groups showed that the highest mean value was observed with Groups IV and II, and the lowest mean value was with Group I. For the /z/ sound, the highest mean value was with Groups IV and V and the lowest mean value was with Group I. For /sh/ sound, the highest mean value was with Groups IV and V and the lowest mean value was with Group II (Figure 1). For /t/ sound, the highest mean value was with Groups IV and III and the lowest mean value was with Group II. For /d/ sound, Groups I and IV showed the highest mean value, and Group II showed the lowest mean value. For /l/ sound, Groups V and I showed the highest mean value and Group III showed the lowest mean value (Figure 2).

The reproduction of rugae area in acrylic denture (Group III) produced highly significant difference with /t/ sound and significant difference with /sh/z/ and /d/ sounds and lowest significant difference with /l/ sound (*P* < 0.05). In the denture with metallic base, Group IV produces highly significant difference with /s/z/sh/t/ and /d/ sounds and significant differences with /l/ sound (*P* < 0.05). In Group V denture with resilient acrylic rugae produces highly significant difference with /z/sh/l/d/ sound and significant differences with /s/t/ sound (*P* < 0.05). Moreover, speaking without denture or with conventional denture without rugae produced the lowest significance with all tested /s/z/sh/t/d/ sounds except /l/ sound (*P* < 0.05).

## 4. Discussion

The present study discusses the changes of the denture factors such as proper rugae shaping and base thickness for adequate tongue space and articulation. If it is determined that the tongue is a possible source of the speech problem, a structural change in the denture should be considered.

Speech problems are frequently reported after complete denture placement mainly expressed as problems with consonants, especially lingopalatal sounds [16]. So, the lingopalatal sounds were analyzed in the present study. Some patients had a remarkable adaptive capacity after repeating 5-6 key words. Analyzed spectrograms are different by frequency (low or high) of emitted sounds related to the denture changes and oral resonance cavity [17]. Specific letters were selected because not all the letters can be verified. According to Sinescu et al., some sounds such as /z/s/t/ and /sh/ are more sensible and more often compromised due to the changes of oral structures and because of the demand for more precise articulation movements. Therefore, these sounds were studied in this work [18].

Attempts to rebuild the rugae palatinae are controversial. It was suggested that the tongue needs surfaces with greater tactile stimulation in analogy to the anatomic structures [17].

The importance of palatal rugae in complete denture construction for proper sound production was revealed by this study. This comes with the agreement of the study, which advocated developing a normal /s/ and /sh/ sounds, and it may be necessary to thicken the incisive papilla region to prevent the jet of air emitted by the median sulcus of the tongue from escaping toward the vault. Bulking of the tongue palatal contact area and the area of the incisive papilla will facilitate proper enunciation and eliminate much of the postinsertion practice period [7, 19].

The metallic dentures selected to provide minimal cross section enhance maximum space for sound production. The essential factor in production of correct /s/ is the proper grooving of the tongue. As the depth of this groove decreased, /s/ is pronounced toward /sh/ as a lisp [20]. Therefore, as in this study, when using metallic denture base, a minimal thickness faced the tongue permitting its proper grooving.

Foti et al. investigate the speech of two edentulous subjects fitted with a complete maxillary prosthesis made of three different palatal materials: aluminium, resin, and stellite. These recordings were tested from the auditory point of view by a series of six listeners (four men and two women) in an anechoic room. The results show that the group fitted with a metal prosthesis (aluminum, stellite) was more intelligible regarding the way they were perceived by others [21].

Evaluation of the speech was performed by acoustic analysis and intelligibility analysis. Reports of acoustic analysis revealed that pronunciation of “s,” “sh,” “t,” and “d” was clearer with rugae incorporated denture than conventional denture. Customized rugae dentures were better than arbitrary rugae dentures. Intelligibility reports showed many substitutional errors with conventional denture. With customized rugae denture speech was clearer [2]. These results agree with the result of this study.

Allen stated that the most widely used procedure to improve denture phonetics is the random thinning of the entire maxillary lingual surface to create more space for the tongue. Such arbitrary removal of acrylic resin from the palatal surface ignores the critical importance of correct palatal contours in the proper formation of sounds [19]. This finding disagrees with the result obtained from this study, because this opinion may be correct in case of lower denture to restore critical tongue space and thinning of acrylic denture may cause fracture.

The results of this study coincide with the result reported by Ravishankar and Singh as they reported that the lingopalatal (anterior) sounds /c/ (soft), /d/t/n/s/z/, and /r/ are formed by the contact of the tip of the tongue with the most anterior part of the palate called the alveolus or the lingual side of anterior teeth. If the denture base in the rugae area is too thick, the /t/ in “tend” sounds like /d/ and the /d/ sounds like /t/ [22].

The patient emotional behavior in relation to a prosthetic treatment can also influence the speech patterns. Patients, who psychologically accepted their dentures, shortly after they started wearing them, also had fewer speech difficulties than those who did not accept the dentures [14] and for this reason, the adaptation period was used in this study.

Therefore, in the adaptation period, a series of speech defects may be noted as hypernasality. As the patient uses his custom-made prosthesis with accurate biomechanical and physiological properties, these complaints decrease and elimination of these problems can be noted over time [23].

The character of speech sounds is difficult to be analyzed and interpreted but it is detected with accuracy by the hearing sense, which is a more sensitive analysis device than well-known handmade devices [24]. This result is not in agreement with the study, as the computerized device is more accurate than hearing sense.

Important investigations of speech sounds are based on the Fourier spectrum and on the short-time Fourier spectrum (spectrogram). The most accurate measurements methods were, as this study suggested, by Jindra et al. [1], Kobayashi et al. [25], and Ögütcen-Toller [26] who chose speech sounds analysis by using standard signal processing methods. The method with the best results was the Fourier method. Its evaluation of the studied phenomena is both qualitatively and quantitatively valuable making the reproducibility of the analysis applicable [17]. So the sample size of three instead of five was used. The Fourier method for the phonetic evaluation was used because its graphic correspondence is simple and facile to translate. The phonetic alterations are considered classic for the human voice and could also be represented even for the higher spectrum. In the present study, the spectrogram was used [17].

## 5. Conclusion

It is recommended to reproduce the rugae area in complete denture because the phonetic quality of complete denture with rugae was superior to the conventional denture. If the denture was too thick in the anterior region, the result would be a faulty /sh/d/z/t/l/ sound. The dentures with metallic base can enhance /s/sh/t/d/ and /z/ sounds. The use of resilient acrylic to reproduce the rugae in complete denture can enhance /z/l/s/sh/t/ and /d/ sounds.

## Figures and Tables

**Table 1:** Comparison between the studied groups according to sounds measured by the maximum energy (frequency, HZ).

Sounds	Group (*n* = 3)	Group I (*n* = 3)	Group II (*n* = 3)	Group III (*n* = 3)	Group IV (*n* = 3)
S (Hz)	1053.7 ± 54.3	1264.2 ± 69.5	1203.6 ± 46.4	1313.4 ± 55.5	1234.8 ± 48.7
Z (Hz)	834.9 ± 55.5	963.6 ± 54.2	1022.5 ± 45.6	1444.0 ± 82.8	1097.0 ± 41.6
Sh (Hz)	1205.8 ± 68.0	1170.6 ± 65.1	1291.0 ± 67.8	1326.9 ± 72.3	1237.0 ± 65.9
T (m/sec.)	4.33 ± 0.1	3.47 ± 0.1	4.47 ± 0.2	4.53 ± 0.1	3.53 ± 0.1
D (m/sec.)	**5.77 ± 0.1**	**1.90 ± 0.1**	**3.83 ± 0.1**	**4.57 ± 0.2**	**4.57 ± 0.2**
L (m/sec.)	**6.97 ± 0.1**	**4.50 ± 0.1**	**4.33 ± 0.2**	**4.40 ± 0.1**	**4.67 ± 0.1**
